# A New *Wnt1* Mutant Rat Model of Osteogenesis Imperfecta and Its Application in AAV9‐Mediated Gene Therapy

**DOI:** 10.1155/humu/7351808

**Published:** 2026-05-21

**Authors:** Shan Li, Xiumin Chen, Yixuan Cao, Mingchen Han, Feifei Guan, Xiuzhi Ren, Huan Mi, Tao Yang, Mei Li, Xiuli Zhao

**Affiliations:** ^1^ Center for Rare Diseases, State Key Laboratory of Complex, Severe, and Rare Diseases, Peking Union Medical College Hospital, Peking Union Medical College and Chinese Academy of Medical Sciences, Beijing, China, pumch.cn; ^2^ State Key Laboratory for Complex, Severe, and Rare Diseases, Department of Medical Genetics, Institute of Basic Medical Sciences, Chinese Academy of Medical Sciences and School of Basic Medicine, Peking Union Medical College, Beijing, China, pumc.edu.cn; ^3^ Department of Molecular Orthopaedics, Beijing Research Institute of Traumatology and Orthopaedics, National Center for Orthopaedics, Beijing Jishuitan Hospital, Capital Medical University, Beijing, China, jst-hosp.com.cn; ^4^ National Human Diseases Animal Model Resource Center, Institute of Laboratory Animal Sciences, Peking Union Medicine College and Chinese Academy of Medical Sciences, Beijing, China; ^5^ Key Laboratory in Science and Technology Development Project of Suzhou, Children′s Hospital of Soochow University, Suzhou, China; ^6^ Department of Endocrinology, Key Laboratory of Endocrinology, National Health and Family Planning Commission, Peking Union Medical College, Chinese Academy of Medical Sciences, Beijing, China, nhfpc.gov.cn

**Keywords:** osteogenesis imperfecta (OI), *Wnt1*, animal model, gene therapy, adeno-associated virus (AAV)

## Abstract

Osteogenesis imperfecta (OI) is a genetically and clinically heterogeneous bone disorder, with more than 20 genes contributing to OI development. Previously, we identified *WNT1* c.620G > A (p.Arg207His) mutation among Chinese patients with autosomal recessive OI (AR‐OI). This study aims at investigating the causative role of *WNT1* deficiency in OI and evaluate whether AAV‐based gene therapy could ameliorate bone abnormalities. We generated and analyzed the Wnt1^R207H/R207H^ rat model. The AAV9‐Wnt1 virus was delivered via direct intraosseous injection into the femoral marrow cavity of the OI rats to evaluate its therapeutic potential. The homozygous Wnt1^R207H/R207H^ rat recapitulated key features of AR‐OI, including fractures, reduced bone mass, growth retardation, decreased survival rate, increased osteoclast numbers, diminished osteoblast function and mineralization capacity, compared with heterozygous and wild‐type littermates. In vitro, *Wnt1* overexpression in osteoblasts promoted osteoblast activity and bone mineralization. Furthermore, AAV9‐Wnt1 treatment in OI rats resulted in significant recovery of bone density and mechanical strength, stimulation of osteoblast activity, suppression of osteoclast activity, and upregulation of Type I collagen expression. Our study demonstrates that *WNT1* c.620G > A (p.Arg207His) is pathogenic, and confirms that AAV‐mediated Wnt1 gene therapy represents a promising strategy for treating OI caused by *WNT1* mutations.

## 1. Introduction

Osteogenesis imperfecta (OI) is a genetically and clinically heterogeneous skeletal disorder characterized by bone fragility and low bone mass [1, 2]. Over 85% of OI cases exhibit autosomal dominant inheritance and are caused by mutations in Type I collagen‐encoding genes *COL1A1* and *COL1A2* [3–5]. In addition, autosomal recessive OI (AR‐OI) is caused by mutations in at least 18 genes (e.g., *P3H1*, *CRTAP*, *PPIB*, *FKBP10*, *BMP1*, *SP7*, *PLOD2*, and *TMEM38B*), which are involved in bone development processes [6, 7]. Although most OI pathogenic genes are involved in collagen synthesis, recent studies have identified several genes not directly involved in collagen synthesis. Among them, *WNT1* mutations cause Type XV OI (OI‐XV), a recessively inherited OI with moderate‐to‐severe phenotypes, primarily by impairing osteoblast differentiation [7, 8]. These findings highlight the complexity of OI pathogenesis. Notably, AR‐OI patients generally exhibit more severe clinical manifestations than those with dominant OI [8].

Currently, there is no curative treatment for OI. Clinical management focuses on improving bone strength, preventing fractures, promoting mobility, and enhancing quality of life [9–12]. While current pharmacological treatments for OI, such as bisphosphonates, can increase bone mineral density (BMD), their efficacy in improving bone strength in certain forms of AR‐OI, including *WNT1*‐related OI, may be limited as they do not directly correct the underlying impairment of osteoblast function [10]. Other therapeutic agents under investigation, for example, teriparatide, denosumab, and transforming growth factor‐beta [TGF*β*] inhibition, have also failed to provide a fundamental cure, especially for severe AR‐OI [12].

Recently, many new therapies have been developed, including cell and gene therapy [13–19]. Stem cell transplantation therapy is aimed at replacing mutant osteoblasts with normal cells. However, its therapeutic potential has been limited primarily by practical challenges, such as the poor engraftment and low survival rate of systemically administered cells in bone tissue [14]. Gene therapy can increase the expression of normal alleles in mutant osteoblasts, potentially ameliorating the severe phenotype [19]. To date, no gene therapy studies have been conducted for OI‐XV, underscoring the need for exploration using relevant animal models. Understanding the functional impact of *WNT1* mutations is essential for developing therapies that restore osteoblast function [20]. Gene therapy represents a rapidly growing field with great potential for treating inherited bone diseases [5].

In a previous Chinese AR‐OI cohort study, we identified *WNT1* as the most frequently mutated gene in AR‐OI cases, with a recurrent mutation of c.620G > A (p.Arg207His) [21]. WNT1 belongs to the WNT family of secreted signaling proteins that are highly evolutionarily conserved—sharing 98% amino acid identity between humans and rats [22]. Both human and mouse genetic studies have demonstrated that canonical Wnt/*β*‐catenin signaling is essential for postnatal bone formation and skeletal development [23].

To elucidate the effect of *Wnt1* mutation on osteoblast morphology and function, we generated a Wnt1^+/R207H^ rat model using CRISPR/Cas9 technology. This study is dedicated to characterizing this homozygous Wnt1^R207H/R207H^ rat model and evaluating whether AAV9‐mediated gene therapy could rescue the OI phenotype. Our results demonstrate that AAV9‐Wnt1 treatment partially restored BMD and significantly increased bone hardness.

## 2. Materials and Methods

### 2.1. Patients

The study was initiated based on genetic findings of a family with severe OI. The proband was diagnosed according to clinical and radiographic features of OI. Genetic analysis identified a homozygous *WNT1* c.620G > A (p.Arg207His) mutation in the proband; both parents were heterozygous carriers. This family is depicted in Figure 1. This study was approved by the Institutional Review Board (IRB) of the Institute of Basic Medical Sciences, Chinese Academy of Medical Sciences, Beijing, China (2020007). Informed consent and authorisation for data publication were obtained from all participants/legal guardians of children under 18. Peripheral blood samples were collected from all available family members. Genetic analysis included Sanger sequencing, conservation analysis, and three‐dimensional protein modeling.

Figure 1Phenotypic characteristics and pathogenicity analysis of a patient with homozygous *WNT1* c.620G > A(p.R207H) mutation. (a) A patient with the homozygous mutation had severe OI clinical manifestations, including ptosis, scoliosis, skeleton deformity, and frequent fractures. Written informed consent and consent to publish the image has been obtained from the parents. (b) The homozygous mutation was found in the patient; both her parents were heterozygous carriers. (c) Multiple‐species sequence alignment shows the evolutionary conservation of position p.R207 in WNT1. (d) The substitution of arginine with histidine eliminates most of the H‐bonds with neighboring amino acids.

**Figure:**
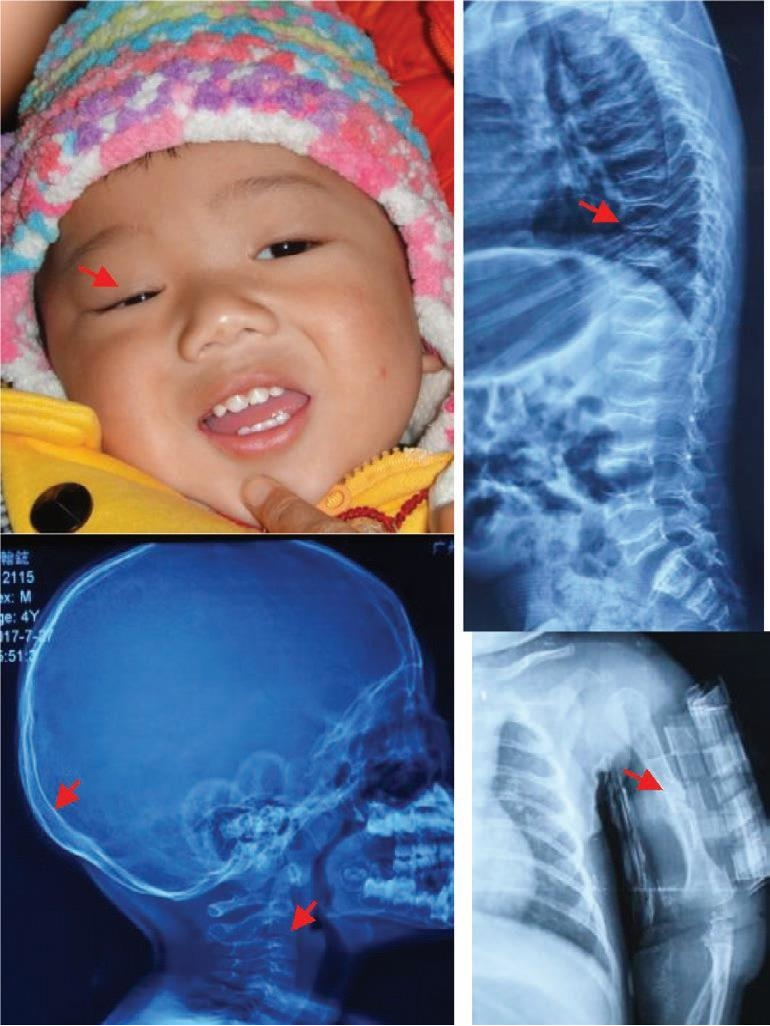
(a)

**Figure:**
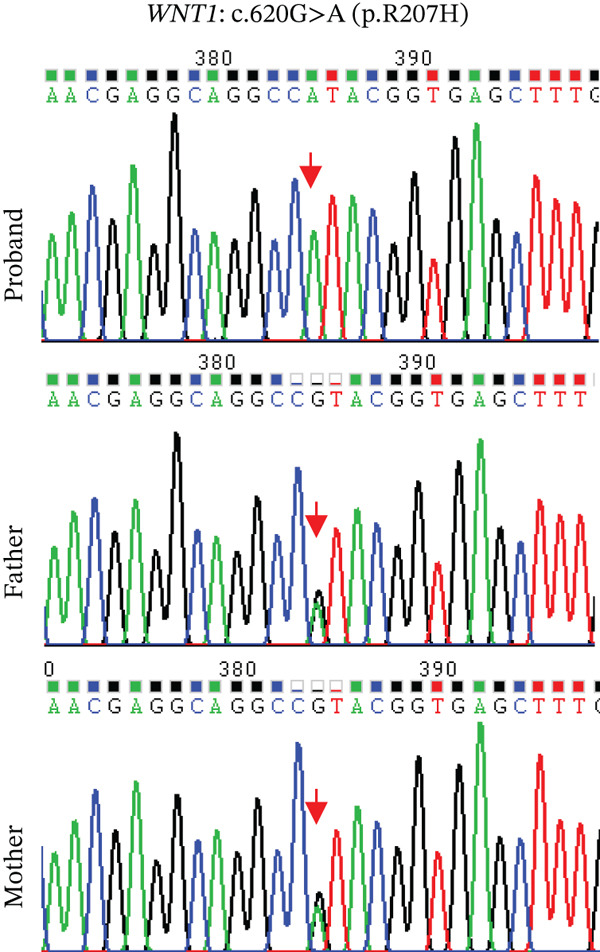
(b)

**Figure:**
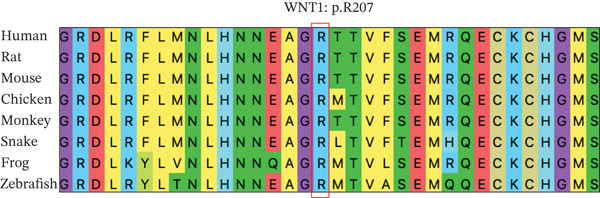
(c)

**Figure:**
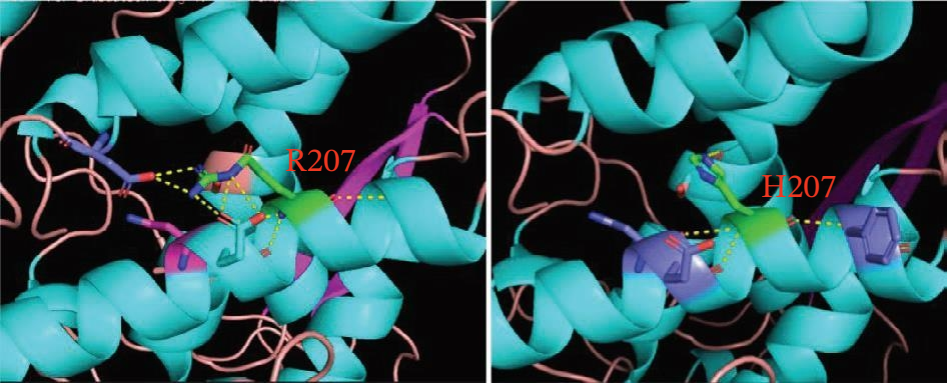
(d)

### 2.2. Animal Model

A rat model carrying the *Wnt1* (NC_005106.4) mutation c.620G > A (p.R207H) was generated using CRISPR/Cas9, based on the homologous recurrent mutation site (c.620G > A, p.Arg207His) identified in human *WNT1* (NM_005430.3). Single‐guide RNA (sgRNAs) oligonucleotides were annealed and cloned into the pUC57‐sgRNA expression vector (#51132, Addgene). The oligonucleotide sequences were synthesized by Thermo Fisher Co. and are listed in Table S1. Briefly, the fertilized eggs were microinjected with a mixture of Cas9 protein, targeting sgRNAs, and oligo donor. Male heterozygous (Wnt1^+/R207H^) rats were mated to heterozygous females to generate homozygous (Wnt1^R207H/R207H^), heterozygous, and wild‐type rats. Genomic DNA was extracted from neonatal rats′ toes using the mouse Direct PCR Kit (Bimake). Genotyping was performed by PCR‐Sanger sequencing. The PCR primers are listed in Table S1. Wild‐type breeding rats were obtained from Beijing Vital River Laboratories Animal Center. All animals were housed under standard conditions with a 12‐h light/dark cycle and ad libitum access to food and water in an assessment and accreditation of laboratory animal care‐accredited SPF animal facility. All animal protocols were approved by the Animal Care and Use Committees of the Institute of Laboratory Animal Science of the Chinese Academy of Medical Sciences (ACUC‐A01‐2020‐002).

### 2.3. Skeletal Analyses

Eight‐week‐old wild‐type, heterozygous (Wnt1^+/R207H^), and homozygous (Wnt1 ^R207H/R207H^) rats were euthanized for skeletal phenotyping using X‐rays, three‐point bending analysis, microcomputed tomography (*μ*CT) analysis, and histological staining.

#### 2.3.1. X‐Ray Examination

Rats were euthanized via CO_2_ asphyxiation, and bone radiography was performed using an Xpert 80 system (Kubtec).

#### 2.3.2. Three‐Point Bending

Mechanical testing was conducted using a computer‐controlled electronic universal material testing machine (HY‐1080). Data were recorded using biomechanical test machine software (mechanical testing M213 pro). Femurs were placed horizontally on the support, and a vertical load was applied at a speed of 0.05 mm/s until fracture. Biomechanical parameters, including bending displacement (mm) and ultimate bending stress (N), were recorded and evaluated.

#### 2.3.3. *μ*CT Analysis

Left femurs were fixed in 4% paraformaldehyde (PFA) for *μ*CT scanning and 3D reconstruction. A transverse section of the distal femur was selected as the object for the scanning, with a 1.0‐mm offset from the proximal growth plate used as the region of interest to analyze the cortical and cancellous bone. BMD, bone volume per total volume (BV/TV), trabecular number (Tb.N), trabecular thickness (Tb.Th), and trabecular separation (Tb.Sp) were analyzed by calculating the morphological parameters with CTAn Version 1.14.4 software (SkyScan; Bruker Corporation).

#### 2.3.4. Histological Analysis

Femur samples were fixed in 4% buffered PFA for 24 h, followed by decalcification in EDTA. The decalcified samples were then embedded in paraffin and sectioned longitudinally. Sections were stained with hematoxylin and eosin (H&E), Masson′s trichrome, and a tartrate‐resistant Acid phosphatase (TRAP) commercial staining kit (TRAP Staining Kit, Servicebio, China). Stained sections were visualized with an optical microscope (NIKON ECLIPSE E100, Nikon, Japan).

### 2.4. Osteoblast Culturing and Staining

Calvarias of 2‐week‐old wild‐type, Wnt1^+/R207H^ and Wnt1^R207H/R207H^ rats were dissected into 1 mm^2^ fragments. Mesenchymal stem cells (MSCs) were isolated via sequential digestion with 0.25% trypsin and 2 mg/mL Collagenase II, and cultured in the basic medium containing DMEM medium (Gibco) supplemented with 10% fetal bovine serum (FBS, Gibco) and 1% penicillin/streptomycin (Gibco). Osteogenic differentiation was induced using basic medium supplemented with 10 mM *β*‐glycerophosphate (Sigma‐Aldrich, St. Louis, Missouri, United States), 10 nM dexamethasone (Solarbio, Beijing, China), and 50 *μ*g/mL l‐ascorbic acid (Solarbio, Beijing, China). Alkaline phosphatase (Solarbio, Beijing, China) staining was performed after 7 days, and mineralization capacity was assessed by alizarin red staining after 14 days.

### 2.5. Osteoclast Culturing and Staining

Bone marrow cells were isolated from the long bones of 2‐week‐old rats. Briefly, the long bones were mashed in a mortar in basic culture medium containing *α*‐MEM (Gibco) supplemented with 5% FBS (Gibco, Grand Island, New York, United States) and 1% penicillin/streptomycin (Gibco). The released whole bone marrow cells were filtered through a 40‐*μ*m filter and cultured at 1.3 × 10^6^ cells/mL in basic culture medium supplemented with 30 ng/mL M‐CSF (R&D Systems, Minneapolis, Minnesota, United States) and 20 ng/mL RANKL (RANKL‐TEC, R&D systems). TRAP staining (Sigma‐Aldrich) was performed after 5 days of culturing following the manufacturer′s instructions.

### 2.6. AAV9‐Wnt1 Treatment of Rats

Based on their genotypes, littermates were divided into two groups: treated OI rats and untreated wild‐type rats. Two‐week‐old Wnt1^R207H/R207H^ rats (*n* = 6) received 20 *μ*L of AAV9‐Wnt1 virus with a titer of 10^13^ vg/mL via femoral puncture in the right femur (OI + Wnt1); the left femur received PBS as an internal control (OI). The femurs of wild‐type rats were employed as the normal controls. Endpoint assessments were performed in a double‐blind manner. Health status and body weight were monitored daily. After 6 weeks of virus injection, animals were euthanized and analyzed for therapeutic effects.

### 2.7. Cell Counting Kit‐8 (CCK8) Assay

Osteoblasts were collected by trypsin digestion, counted, and seeded at a density of 5 × 10^3^ cells/well in a 96‐well plate. For analysis, 10 *μ*L CCK‐8 (Dojindo, Shanghai, China) was added to each well, and the plate was incubated at 37°C for 3 h until formazan was formed. The absorbance was recorded at 450 nm using an enzyme‐linked reaction analyzer (FlexStation 3, molecular devices). The experiment was designed with six time points, spanning from the first day (D1) to the sixth day (D6) after resuscitation, with one test conducted per day.

### 2.8. Western Blotting

Proteins were extracted using RIPA lysis buffer (P0013B, Beyotime, China), separated by 10% SDS‐PAGE, and transferred to PVDF membranes (IPVH00010, Millipore, United States). The antibodies included pro‐*α*1(I) (Abcam, 1:1000), Wnt1 (Abcam, 1:1000), Gapdh (Transgen, 1:2000), and HRP‐labeled goat antirabbit antibodies (CST, 1:2000).

### 2.9. Quantitative PCR (qPCR)

Total RNA was extracted using the Bone Tissue RNA Rapid Extraction Kit (Genenode Inc., China, 312452AX) according to the manufacturer′s instructions and quantified with a NanoPhotometer (Implen Inc. Germany). cDNA was prepared from 1 *μ*g RNA with the PrimeScript RT Reagent Kit and gDNA Eraser (TaKaRa, Dalian, China). qPCR was performed in a 20‐*μ*L reaction volume containing TB Green Premix Ex Taq II (TaKaRa, Dalian, China, No. RR820L) and gene‐specific primers (Table S1). Amplification and quantification were performed with a qPCR analyzer (Bio‐Rad, United States) using the following qPCR conditions: preincubation at 95°C for 3 min, followed by 45 cycles of denaturation at 95°C for 5 s, annealing at 60°C for 30 s, and extension at 72°C for 30 s. Melting curves were analyzed to ensure the amplification of single PCR products. *Gapdh* gene was used as the endogenous control, and fold changes were calculated using the 2‐^*ΔΔ*Ct^ method.

### 2.10. Statistical Analysis

Data were analyzed using GraphPad Prism 9.0 and are presented as means ± standard deviations. A one‐way analysis of variance (ANOVA) was conducted to evaluate differences among multiple groups. For comparisons between two groups, an independent‐samples *t*‐test was applied. A *p* value ≤0.05 was considered significant (^*^, *p* < 0.05; ^**^, *p* < 0.01; ^***^, *p* < 0.001, ^****^ *p* ≤ 0.0001), whereas a *p* ≥  0.05 was considered not significant.

## 3. Results

### 3.1. Identification of a Homozygous WNT1 c.620G > A (Arg207His) Mutation

In this study, we collected a patient who exhibited severe OI clinical manifestations, including ptosis, scoliosis, skeletal deformity, and frequent fractures (Figure 1a). Genetic analysis identified a homozygous *WNT1* c.620G > A (p.Arg207His) mutation in the proband; both parents were heterozygous carriers.(Figure 1b). Sequence alignment among multiple species showed the highly evolutionary conservation of p.R207 in protein WNT1 (Figure 1c). According to the SWISS‐MODEL prediction, the substitution of arginine to histidine would likely affect the conformation and charge properties of the protein and would eliminate most of the H‐bonds with neighboring amino acids (Figure 1d). These findings support the likely pathogenicity of the c.620G > A (p.Arg207His) mutation.

### 3.2. Generation and Characterization of Wnt1^+/+^, Wnt1^+/R207H^, and Wnt1^R207H/R207H^ Rats

We constructed the mutant Wnt1 rat model by targeted knock‐in of the R207H mutation using CRISPR/Cas9 gene editing (Figure 2a,b). Of the 198 rats generated, 16.2% were homozygous (Wnt1 ^R207H/R207H^, referred to as OI rats), among which 31.25% exhibited postnatal lethality before 3 weeks of age (Table 1). One OI rat displayed unilateral ptosis in the right eye (Figure S1), resembling clinical manifestations in WNT1‐related OI patients. Radiographic analysis revealed severe osteopenia in OI rats, characterized by increased radiolucency in both trabecular and cortical bones (Figure 2c). OI rats also showed significant growth retardation, as reflected by reduced weight gain compared with their wild‐type littermates (Figure 2d). Histological analysis indicated decreased collagen fiber content and fibrosis in OI rats (Figure 2e). Spontaneous tibial fractures accompanied by callus formation and deformed femurs were also observed (Figure 2f).

Figure 2Generation and characterization of a rat model carrying the *Wnt1* c.620G > A(p.R207H) mutation. (a) Schematic diagram of animal model construction using CRISPR/Cas9, including rat′s homologous sequence to *WNT1* mutation region in the OI patient. (b) Sanger sequencing results for Wnt1 in the Wnt1^+/+^, Wnt1^+/R207H^, and Wnt1^R207H/R207H^ rats. The red arrow indicates the mutated nucleotide. (c) Whole body radiography of wild‐type, heterozygote, and homozygous OI rats (2 weeks old). Homozygous OI rats were smaller and had lower BMD than littermates of the wild‐type rats and heterozygotes. (d) The weight was measured every 10 days from 15 days to 55 days of age. OI rats had lower body weight than wild‐type rats and showed growth retardation during the whole growth process. (e) Masson‐stained longitudinal sections of femurs of wild‐type and OI rats at 6 weeks of age. The collagen fiber content and the degree of fibrosis in OI rats were significantly lower than those in wild‐type rats. (f) Spontaneous fracture in the tibia evidenced by callus formation and (g) by x‐ray at 8 weeks of age. ^***^, *p* < 0.001; ^****^, *p* < 0.0001; WT: wild type.

**Figure:**
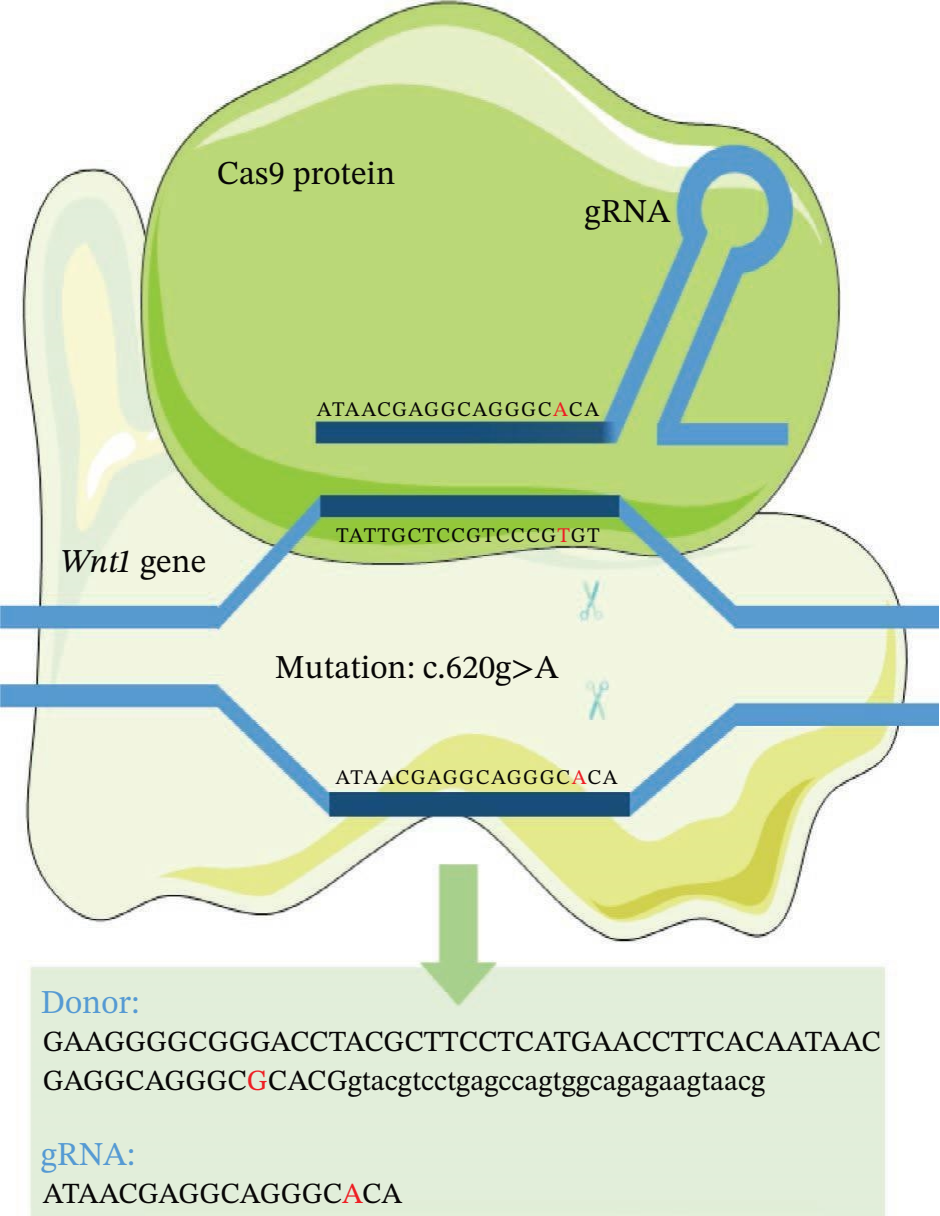
(a)

**Figure:**
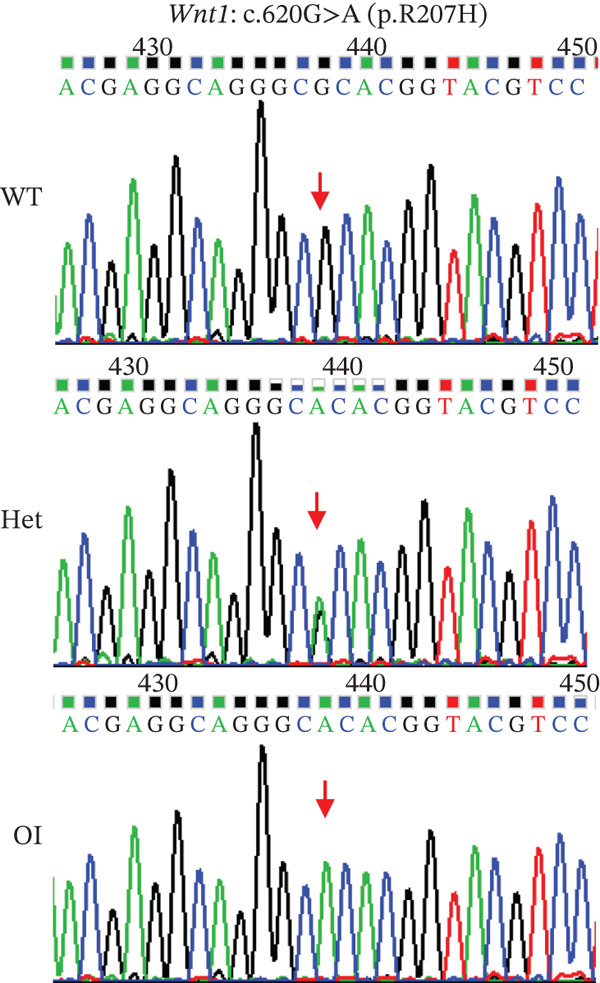
(b)

**Figure:**
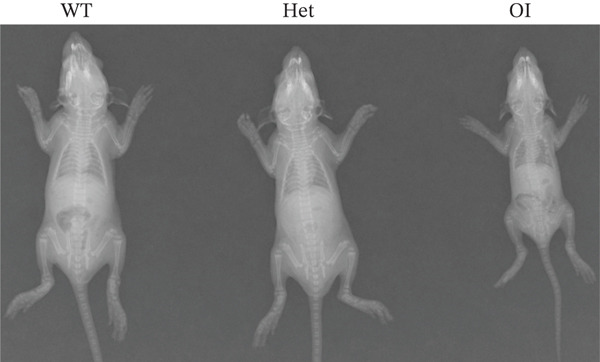
(c)

**Figure:**
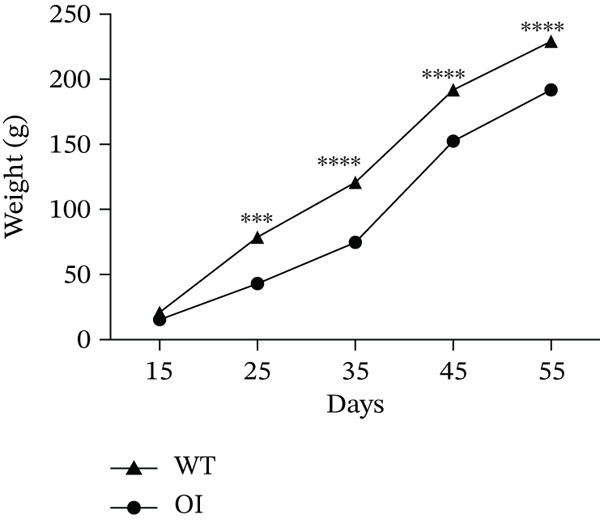
(d)

**Figure:**
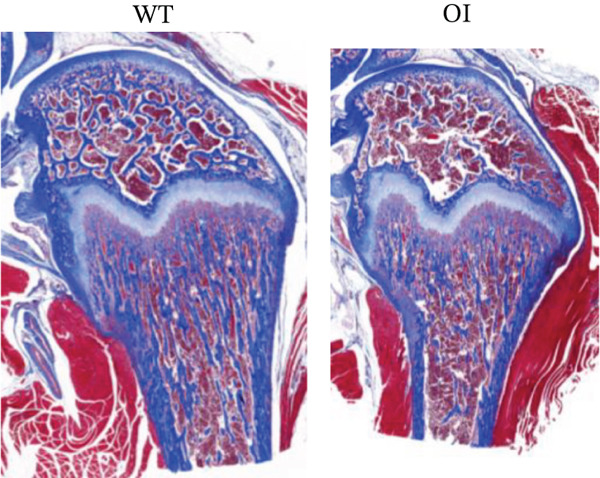
(e)

**Figure:**
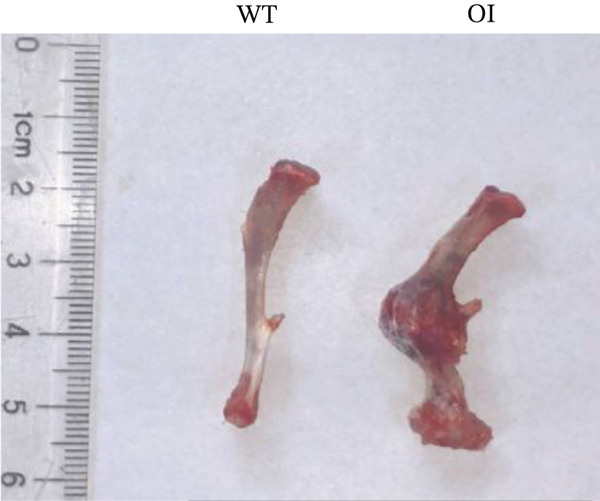
(f)

**Figure:**
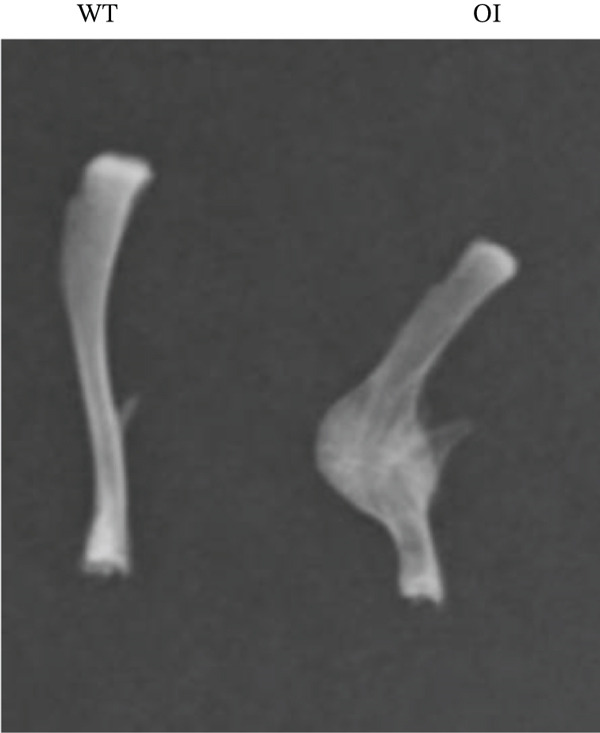
(g)

**Table 1:** Analysis of genotype and postnatal lethality rate of wild‐type, heterozygous and homozygous (OI) rats.

Genotype	Number of rats	Birth rate	Postnatal lethality (%)
WT	46	23.2	0
Heterozygous	120	60.6	0
OI	32	16.2	31.25
Total	198	100	

### 3.3. Micro‐CT and Biomechanical Analysis

*μ*CT of long bones showed that OI rats had reduced trabecular bone volume (BV/TV) and thinner cortical bone (Figure 3a). BV/TV decreased by approximately 50% compared with wild‐type controls (Figure 3b). OI rats also exhibited significantly lower Tb.N, reduced Tb.Th, and increased Tb.Sp. The thickness of the cortical bone (Cort.Th) in the OI rats was also significantly decreased. Three‐point bending tests of femurs from 8‐week‐old rats demonstrated that OI rats had markedly reduced maximum load and stiffness, indicating impaired bone strength and resistance to mechanical stress (Figure 3c).

Figure 3Reduced bone mass in OI rats. (a) The 3D reconstructed images of cortical (above) and trabecular (below) bones of the femurs from 8‐week‐old wild‐type and OI rats. The femur density of OI rats was lower than that of wild‐type rats. (b) The statistical analysis of trabecular microarchitecture parameters. Bone volume/tissue volume (BV/TV), trabecular thickness (Tb.Th, mm), Tb.N (1/mm), trabecular spacing (Tb.Sp, mm), and cortical bone thickness (Cort.Th). Compared with wild‐type femurs, BV/TV, Tb.Th, Tb.N, and Cort.Th were significantly decreased in OI rats, whereas Tb.Sp was significantly increased, suggesting femur bone quality was impaired in OI rats. (c) The biophysical properties were reflected by maximum load and stiffness. The maximum endurance and flexural length of the femurs from OI rats were reduced compared with those from wild‐type rats. ∗, *p* < 0.05; ∗∗, *p* < 0.01; ∗∗∗^*^, *p* < 0.0001. WT: wild type

**Figure:**
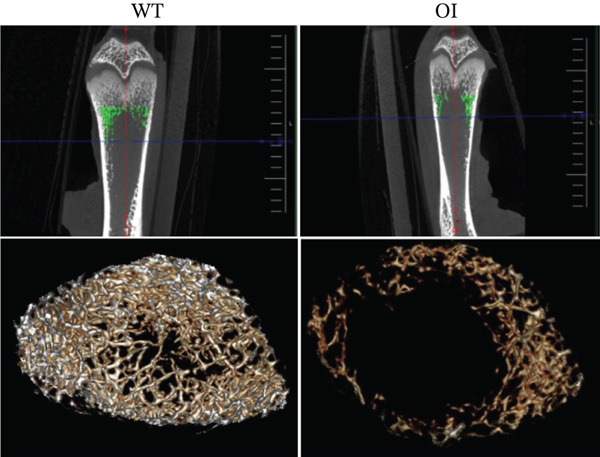
(a)

**Figure:**
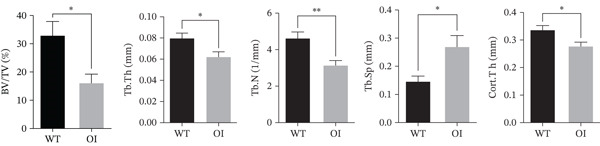
(b)

**Figure:**
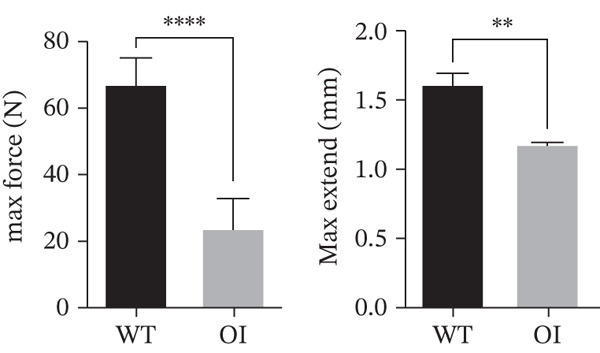
(c)

### 3.4. Activity of Osteoblasts and Osteoclasts In Vitro

CCK8 assays revealed no significant difference in osteoblast proliferation among wild‐type, heterozygous, and homozygous rats (Figure 4a). However, ALP staining indicated impaired osteogenic differentiation in OI rats, and alizarin red staining indicated reduced mineralization capacity (Figure 4b). Trap staining was used to detect the activity of osteoclasts, demonstrating increased osteoclastogenesis in OI rats (Figure 4b). Compared with wild‐type samples, the femurs of the OI rats showed significantly decreased mRNA expression of osteoblast markers, including alkaline phosphatase (*Alp*), runt‐related transcription factor 2 (*Runx2*), osteopontin (*Opn*), and osteocalcin (*Ocn*) (Figure 4c). In contrast, mRNA expression levels of osteoclast markers, including *Trap*, receptor activator of NF‐*κ*B (*Rank*), osteoclast‐associated lg‐like receptor (*Oscar*), and nuclear factor of activated T‐cells 1 (*Nfatc1*) were significantly upregulated (Figure 4d).

Figure 4Comparison of cell proliferation, osteoblasts, and osteoclasts in rats with different genotypes. (a) D1 to D6 represent six time points for cell proliferation analysis, and no difference was found in cell proliferation among rats of the three genotypes. (b) ALP staining and Alizarin Red staining revealed that osteogenesis and mineralization in OI rats were significantly diminished compared to wild‐type rats, and TRAP staining showed that the osteoclast activity of OI rats was stronger than that in wild type rats. (c, d) The qPCR results indicated that, compared with wild‐type rats, osteoblast markers (*Alp*, *Runx2*, *Opn*, and *Ocn*) were significantly decreased in OI rats, whereas osteoclast markers (*Trap*, *Rank*, *Oscar*, and *Nfatc1*) were significantly increased in the OI group. ∗, *p* < 0.05; ^**^, *p* < 0.01; *n* = 6. WT: wild type.

**Figure:**
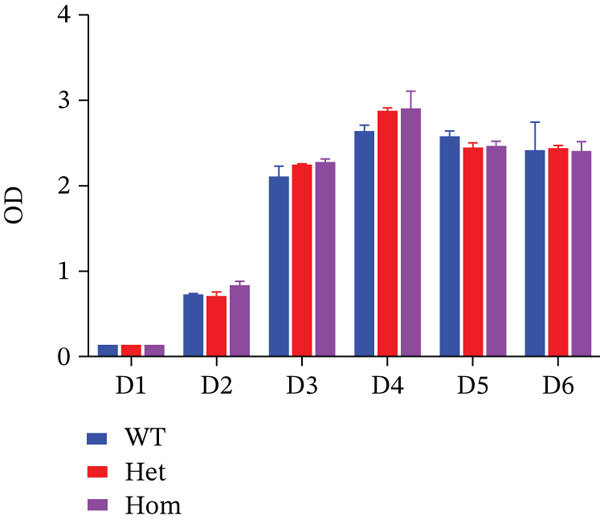
(a)

**Figure:**
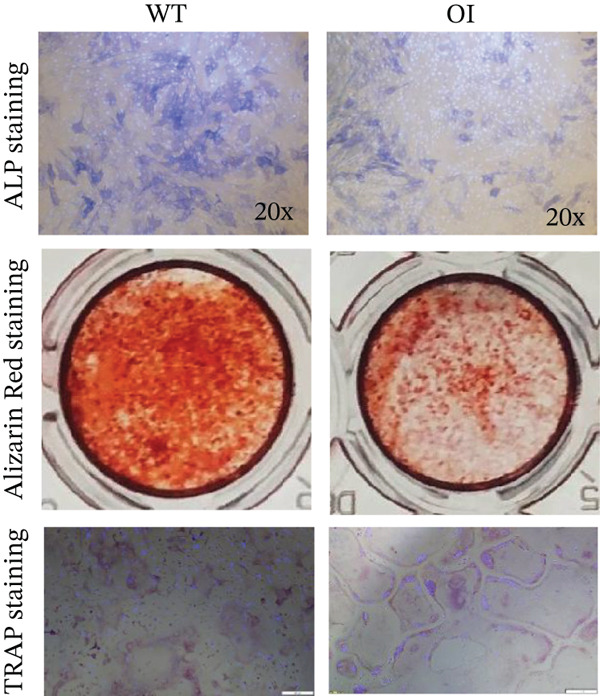
(b)

**Figure:**
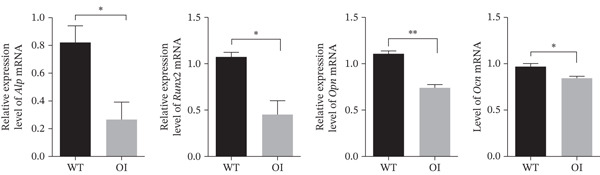
(c)

**Figure:**
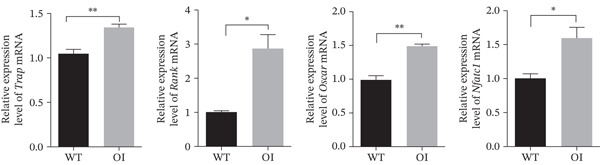
(d)

### 3.5. Overexpression of Wnt1 in Osteoblasts In Vitro

Transfection of AAV9‐Wnt1 into osteoblasts derived from OI rats did not significantly alter collagen Type I (COL1) protein levels (Figure 5a), suggesting that the Wnt1 mutation does not directly affect collagen expression. However, ALP and Alizarin Red staining showed that *Wnt1* overexpression significantly enhanced osteogenic differentiation and mineralization capacities compared with untransfected OI osteoblasts (Figure 5b).

Figure 5Effects of *Wnt1* overexpression in OI rat osteoblasts. (a) The levels of type I collagen and Wnt1 in the OI rat group were elevated compared with those in the wild‐type group, yet there were no significant disparities between the OI rat group and the *Wnt1* overexpression group. (b) ALP staining and Alizarin Red staining revealed that osteogenesis and mineralization in the OI group were diminished compared with the wild‐type; overexpression of *Wnt1* showed a significant rescue on osteogenesis and mineralization, approaching or even surpassing the levels of the wild‐type. COL1: type I collagen; WT: wild type.

**Figure:**
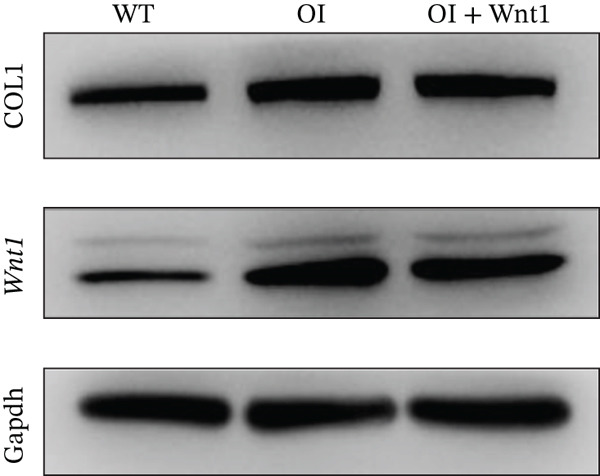
(a)

**Figure:**
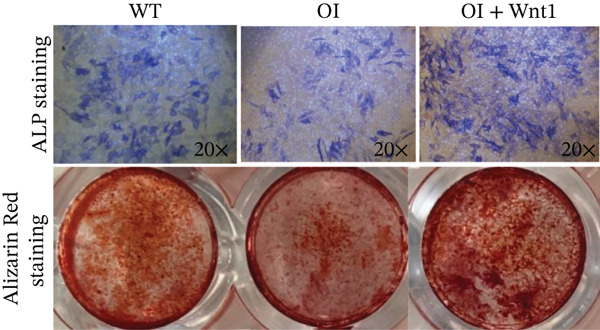
(b)

### 3.6. In Vivo Rescue of Skeletal Phenotypes by AAV9‐Wnt1

#### 3.6.1. Femoral Morphology and Mechanical Properties

After 6 weeks of AAV9‐Wnt1 virus injection, bilateral femurs were analyzed. We found that the femurs from the AAV9‐Wnt1 treated group exhibited a significant increase in cortical bone thickness and demonstrated significantly improved biomechanical properties, including increased maximum load and energy to failure, compared with the PBS‐treated OI controls (Figure 6a), so the AAV‐Wnt1 treatment rescued the OI manifestation.

Figure 6Assessment of AAV9‐Wnt1 treatment in OI rats. (a) Macroscopic appearance. AAV‐Wnt1 treatment increased thickness and straightness of the femurs. (b) *μ*CT reconstructed image of the trabecular bones. AAV9‐Wnt1 virus penetrated the femurs and increased bone mineral density. (c) Statistical analysis of trabecular microarchitecture parameters (for each group). Bone volume/tissue volume (BV/TV), bone surface area/tissue volume (BS/TV), trabecular thickness (Tb.Th), Tb.N (1/mm), trabecular spacing (Tb.Sp, mm), and cortical bone thickness (Cort.Th). AAV9‐Wnt1 virus treatment significantly increased BV/TV; other parameters such as Tb.N, Tb.Th, Cort.Th, BS/TV, and Tb.Sp showed improvement without reaching significance. (d–e) The biochemical properties were reflected by maximum load and stiffness. When the AAV9‐Wnt1 virus penetrated the femur, the maximum bending force increased; however, the maximum bending distance did not change significantly. ∗, *p* < 0.05; ∗∗, *p* < 0.01; ^***^<0.001 ; WT: wild type.

**Figure:**
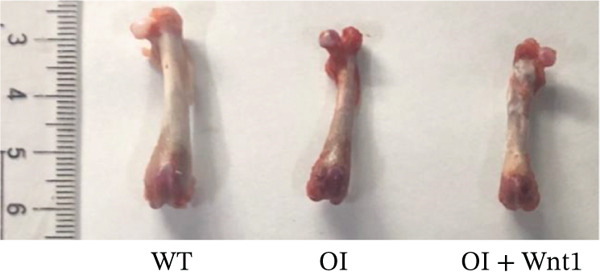
(a)

**Figure:**
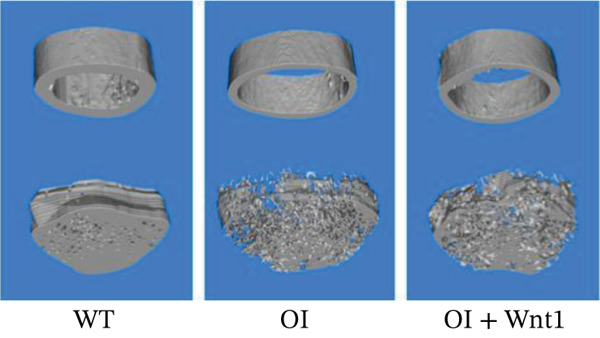
(b)

**Figure:**
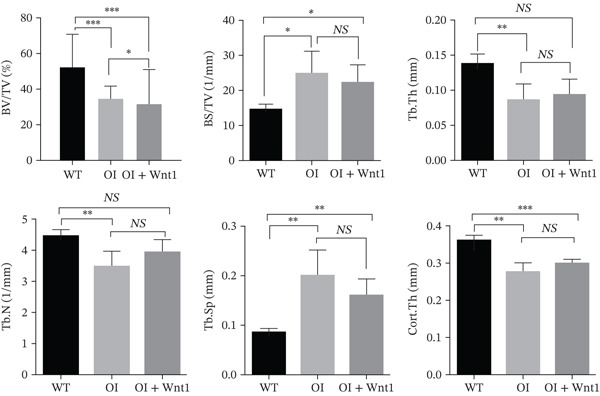
(c)

**Figure:**
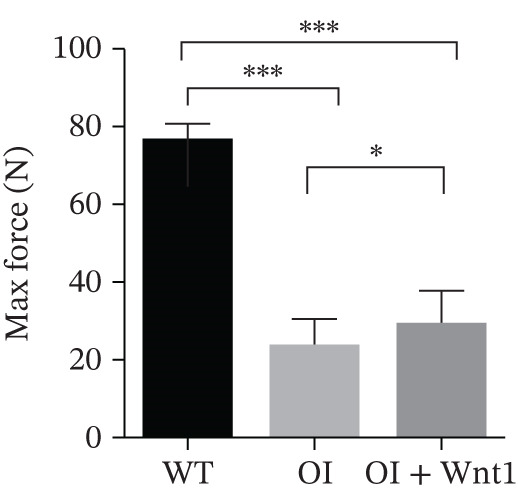
(d)

**Figure:**
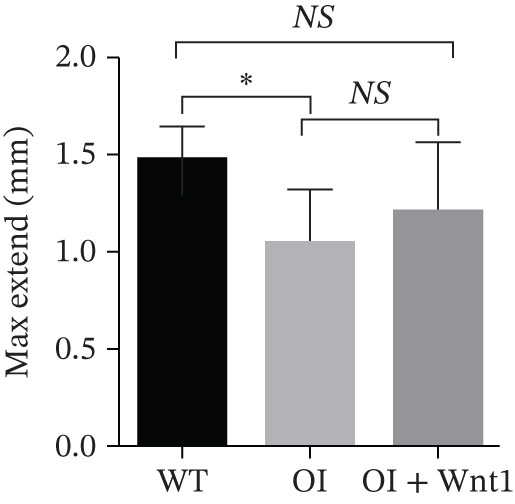
(e)

#### 3.6.2. Bone mineral density by *μ*CT

*μ*CT analysis revealed significantly lower trabecular and cortical bone density in OI rats compared with wild‐type. AAV9‐Wnt1 treatment increased BMD and BV/TV in OI rats (Figure 6b). Improvements were also observed in Tb.N, Tb.Th, Cort.Th, BS/TV, and Tb.Sp, though these changes did not reach statistical significance (Figure 6c,*p* > 0.05). These findings suggest that AAV9‐Wnt1 treatment partially restores bone volume in OI rats.

#### 3.6.3. Femur stress analysis

Three‐point bending tests indicated that AAV9‐Wnt1 treatment increased the maximum force required for fractures in femurs compared with the OI group. The maximum extension also showed an increasing trend, though the difference was not statistically significant (Figure 6d,e). These results demonstrate that AAV9‐Wnt1 enhances bone strength in OI rats.

#### 3.6.4. Histological analysis of the bones

Masson′s trichrome staining showed decreased collagen fiber content in OI rats, which was increased after AAV9‐Wnt1 treatment (Figure 7a). TRAP staining indicated elevated osteoclast numbers along trabecular surfaces in OI rats, which were reduced following treatment (Figure 7b). These results confirm that AAV9‐Wnt1 promotes collagen deposition and suppresses osteoclastogenesis.

Figure 7Histochemical results of AAV9‐Wnt1 treatment of OI rats. (a) Masson staining of samples from the OI + Wnt1 group showed a significant increase in collagen fibers and tissue morphology. (b) TRAP staining of femurs isolated from 8‐week‐old male wild‐type, OI, and OI + Wnt1 rats. Osteoclast numbers were reduced after treatment. (c) qPCR analysis of osteoclast markers (*Rank*, *Oscar*, *Nfatc1*) in femoral tissue. Expression levels were reduced in the OI + Wnt1 group. ∗, *p* < 0.05; ∗∗, *p* < 0.01; ^***^< 0.001; WT: wild type.

**Figure:**
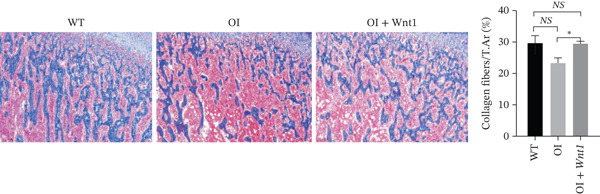
(a)

**Figure:**
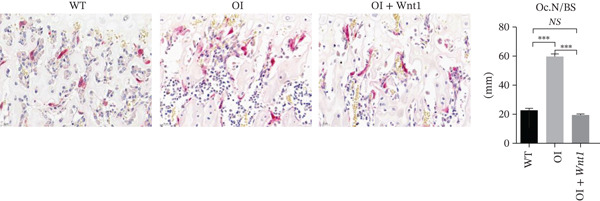
(b)

**Figure:**
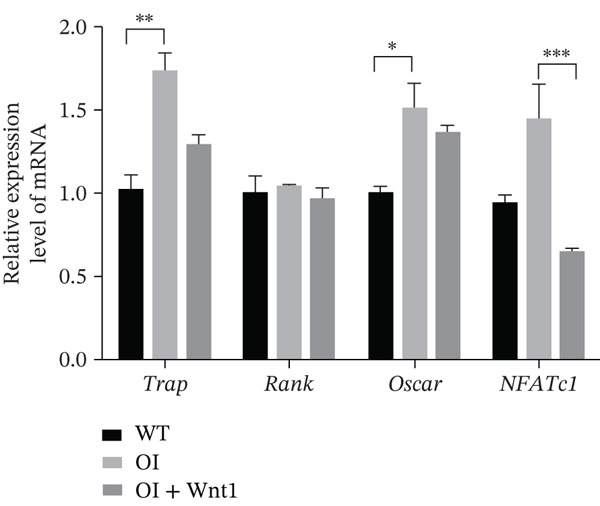
(c)

#### 3.6.5. Expression of osteoclast markers

Although the mRNA expression levels of key osteoclast genes (*RANK*, *OSCAR*, and *NFATc1*) in the rAAV9‐Wnt1‐treated group showed a downward trend that was lower than that in the OI group, statistical analysis indicated that this difference did not reach a significant level (Figure 7c). This initial finding is worthy of further verification in future studies through increasing the sample size or optimizing the dosing regimen.

## 4. Discussion

OI is a congenital skeletal disorder characterized by reduced bone mass and increased fracture risk, with heterogeneous inheritance patterns including autosomal recessive forms (AR‐OI). AR‐OI, particularly the WNT1‐related Type XV, presents with severe manifestations that respond poorly to conventional pharmacological and surgical interventions. These limitations highlight the urgent need for targeted therapeutic strategies. Recombinant adeno‐associated virus (rAAV) is a single‐stranded linear DNA vector known for its low toxicity, low immunogenicity, and sustained transgene expression [24]. Since its discovery in 1965, rAAV has been applied in clinical settings for several genetic diseases and is currently regarded as one of the most promising gene delivery systems [25, 26].

In a previous study involving a Chinese cohort of 74 AR‐OI patients, we identified *WNT1* as the most frequently mutated gene, accounting for 40.54% of cases [21]. There were 30 probands caused by *WNT1* mutations, with eight alleles of the c.620G > A (Arg207His) mutation identified and three probands representing homozygotes with this mutation [21]. Homozygotes with this mutation have severe clinical manifestations, including ptosis, scoliosis, skeletal deformities, and frequent fractures. To determine the pathogenicity of this mutation and explore gene therapy for OI, we generated a rat model by gene editing to introduce the missense mutation R207H. This rat model successfully demonstrated key features of OI‐XV (e.g., short stature, reduced bone density, and growth retardation). The homozygous mutant rats showed a mortality rate of 31.25% within the first 3 weeks, along with fractures, decreased bone density, and neurological abnormalities including ptosis.

Joeng et al. reported homozygous mice with a spontaneous 1‐bp deletion (c.565delG) in the *Wnt1* gene that showed low BMD, swinging walk, rotational behavior, cerebellar defects, and severe neurological disease manifestations, though high mortality was not observed [27]. More recently, two independent research teams also generated mouse models with *Wnt1* mutations showing skeletal defects [28, 29]. Yorgan et al. showed that Wnt1^+/R235W^ and Wnt1^R235W/R235W^ mice were born at the expected Mendelian ratio without postnatal lethality or obvious nonskeletal phenotypes [29]. Interestingly, the phenotype of our rat model differs from that of Wnt1^R235W/R235W^ mice, a mutation associated with early‐onset osteoporosis (EOOP) suggesting that different mutations may lead to distinct phenotypes. In contrast to Wnt1^R235W/R235W^ mice, the newly established Wnt1^G177C/G177C^ model of mouse displays not only a skeletal phenotype with earlier onset but also a high incidence of skeletal fractures [28]. Although two OI‐XV children with the homozygous G177C mutation have been identified with a severe neurological phenotype and brain atrophy, the Wnt1^G177C/G177C^ mice developed a pronounced bone phenotype without the brain abnormalities previously described for Wnt1^−/−^ or Wnt1^sw/sw^ mice [28]. All the studies using *Wnt1* animal models indicate a crucial role for Wnt1 in bone remodeling.

AAV‐based gene therapy is gaining popularity owing to its excellent safety profile and effective therapeutic outcomes in several diseases [24]. To date, two AAV therapeutics are currently approved by the FDA, including voretigene neparvovec (Luxturna) for RPE65‐mediated inherited retinal dystrophy, and onasemnogene abeparvovec (Zolgensma) for spinal muscular atrophy [25]. The full‐length *WNT1* cDNA is 1113 bp, and is suitable for carrying and transporting in a rAAV vector [30–36]. In this study, we constructed the rAAV9‐Wnt1 virus vector, injected it into the femurs of Wnt1‐OI rats, and found that there was an increased BMD in the virus‐treated femurs of the OI rats. Our study represents the first gene therapy study in the OI‐XV model by a viral vector system to overexpress the target gene. Both in vitro and in vivo results demonstrated that *Wnt1* overexpression enhanced bone volume, promoted bone formation and mineralization, reduced bone resorption, and partially restored bone microstructure (Figure 5, Figure 7). These findings suggest that rAAV9‐mediated Wnt1 delivery may ameliorate skeletal abnormalities in Wnt1‐related OI and hold therapeutic potential for this condition. A similar strategy could be extended to other autosomal recessive disorders, provided the pathogenic gene′s coding sequence is within the packaging capacity of rAAV (less than ∼4.7 kb). Although the rAAV9‐Wnt1 virus increased bone density, the effects were not significant particularly in cortical thickness and Tb.N. Future studies should focus on improving the structure of the viral vector, intervention time, and injection dose of virus to enhance therapeutic outcomes.

In summary, we established a novel rat model carrying a recurrent *WNT1* mutation identified in the Chinese AR‐OI population, which accurately simulates the clinical phenotypes of OI‐XV. We further demonstrated that rAAV‐mediated *Wnt1* gene therapy partially rescues bone abnormalities in this model. Our findings support the potential of AAV‐based gene therapy as a viable treatment strategy for OI and provide a foundation for future clinical translation.

## Author Contributions

S.L., X.C., and Y.C. performed the main experiments. M.H. and F.G. performed experiments and analyzed data. H.M. and T.Y. provided and debugged experimental tools. S.L., X.C., and Y.C. wrote the manuscript. M.L. instructed the phenotype analysis of the animal model. X.Z. conceived the study and supervised this research. S.L., X.C., and Y.C. contributed equally to this study.

## Funding

This study was supported by National Key Research and Development Program of China (10.13039/501100012166, 2022YFC2703700); CAMS Innovation Fund for Medical Sciences (2021‐I2M‐1‐051); National Natural Science Foundation of China (10.13039/501100001809, 81472053, 82000846); Beijing Municipal Natural Science Foundation (10.13039/501100005089, 7244286); National High Level Hospital Clinical Research Funding (2025‐PUMCH‐E‐001).

## Disclosure

All the authors critically reviewed and approved the final version of the manuscript.

## Ethics Statement

The studies involving human participants were reviewed and approved by the Institutional Review Board (IRB) of the Institute of Basic Medical Sciences, Chinese Academy of Medical Sciences, Beijing, China (2020007). All animal protocols were approved by the Animal Care and Use Committees of the Institute of Laboratory Animal Science of the Chinese Academy of Medical Sciences (ACUC‐A01‐2020‐002).

## Conflicts of Interest

The authors declare no conflicts of interest.

## Supporting Information

Additional supporting information can be found online in the Supporting Information section.

## Supporting information


**Supporting Information 1** Table S1: Primers used in this study.

**Supporting Information 2** Figure S1: The right eye of the OI rat showed ptosis symptoms similar to those of the proband with WNT1 mutation.

## Data Availability

Requests to access the datasets should be directed to the corresponding author.
