# Supplementary material for: A New Wnt1 Mutant Rat Model of Osteogenesis Imperfecta and Its Application in AAV9‐Mediated Gene Therapy
Source: Hum Mutat. 2026 May 21;2026:7351808. doi: 10.1155/humu/7351808 (PMC13191825; doi:10.1155/humu/7351808)
Supplement: Supplementary file 1 — Supporting Information 1 Table S1: Primers used in this study. [file HUMU-2026-7351808-s001.docx]

**Table S1 Primers used in this study**

(1) The oligonucleotides used for sgRNA expression vectors

| R-Wnt1 -gRNA-top | 5’- TAGGATAACGAGGCAGGGCGCA-3’ |
| --- | --- |
| R-Wnt1 -gRNA-down | 5’- AAACCCGTTCCTCACTAGGCTC-3’ |
| R-Wnt1-oligo | 5’-gaaggggcgggacctacgcttcctcatgaaccttcacaataacgaggcagggcAcacggtacgtcctgagccagtggcagagaagtaacg-3’ |

(2) The primers for amplifying and sequencing CRISPR/Cas9-induced mutations

| Name | Sequence (5’-3’) | Amplicon |
| --- | --- | --- |
| R-Wnt1 - F1 | 5’gaagtatggtgaggagattattcgtg-3’ | 737 bp |
| R-Wnt1 - R1 | 5’AGGAGAGAGGAAGTGCACAATG-3’ |  |

(3) Primers for gene expression analysis

| Gene | Primer（5’to 3’） | |
| --- | --- | --- |
| *Gapdh* | F: GGCAAGTTCAACGGCACAG | R: CGCCAGTAGACTCCACGACAT |
| *Alp* | F: ACGAGGTCACGTCCATCCT | R: CCGAGTGGTGGTCACGAT |
| *Runx2* | F: CACAAGTGCGGTGCAAACTT | R: AATGACTCGGTTGGTCTCGG |
| *Opn* | F: AGCAAGAAACTCTTCCAAGCAA | R: GTGAGATTCGTCAGATTCATCCG |
| *Ocn* | F: GGGCCTTTGCTTTCCATATT | R: CAGTGGCATTAACCAACACG |
| *NFATc1* | F: ACCACCCAGTACACCAGCTC | R: GGGCTGTCTTTCGAGACTTG |
| *Oscar* | F: TCTCCAGGGGCCCAAATCTA | R: TTTCAGGCTGAGTACCGTGG |
| *Trap* | F: AGCGATCACCGCTTCTGTTC | R: CAGCACCATCCACGTATCCA |
| Rank | F: ACCTGTCTTCTAAATGCACTC | R: CTTGCCTGCATCACAGACTT |
