# Supplementary material for: A New Wnt1 Mutant Rat Model of Osteogenesis Imperfecta and Its Application in AAV9‐Mediated Gene Therapy
Source: Hum Mutat. 2026 May 21;2026:7351808. doi: 10.1155/humu/7351808 (PMC13191825; doi:10.1155/humu/7351808)
Supplement: Supplementary file 2 — Supporting Information 2 Figure S1: The right eye of the OI rat showed ptosis symptoms similar to those of the proband with WNT1 mutation. [file HUMU-2026-7351808-s002.ppt]

## Slide 1
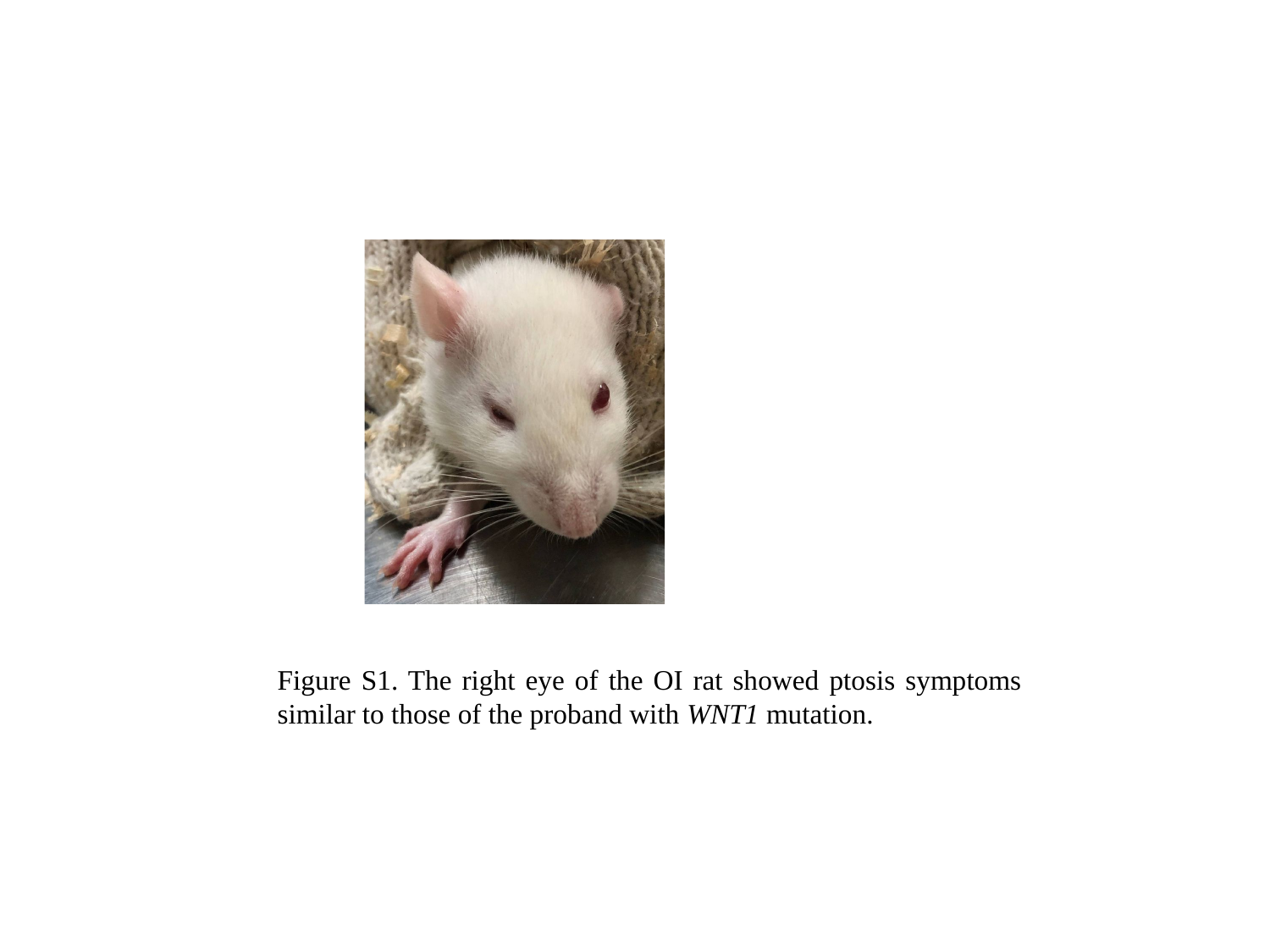

Figure S1. The right eye of the OI rat showed ptosis symptoms similar to those of the proband with WNT1 mutation.
